# First-line tremelimumab plus durvalumab and chemotherapy *versus* chemotherapy alone for metastatic non-small cell lung cancer: a cost-effectiveness analysis in the United States

**DOI:** 10.3389/fphar.2023.1163381

**Published:** 2023-07-20

**Authors:** Wenjie Liu, Gengwei Huo, Peng Chen

**Affiliations:** Department of Thoracic Oncology, Tianjin Medical University Cancer Institute and Hospital, Key Laboratory of Cancer Prevention and Therapy of Tianjin, National Clinical Research Center for Cancer, Tianjin’s Clinical Research Center for Cancer, Tianjin, China

**Keywords:** cost-effectiveness, tremelimumab, durvalumab, non-small cell lung cancer, Markov model, POSEIDON

## Abstract

**Importance:** In the open-label phase III POSEIDON randomized clinical trial (RCT), a limited course of tremelimumab plus durvalumab and chemotherapy (T + D + CT) indicated in the first-line treatment of metastatic non-small cell lung cancer (mNSCLC), progression-free survival, and overall survival (OS) were substantially improved without significant additional tolerance burden compared to chemotherapy (CT). However, given the high cost of T + D + CT, its value needs to be evaluated in terms of both potency and cost.

**Objective:** To evaluate the cost-effectiveness of T + D + CT *versus* CT in individuals with previously untreated mNSCLC from a U.S. payer perspective.

**Design, setting, and participants:** A three-state Markov model was adopted to weigh the lifetime costs and effectiveness of T + D + CT *versus* CT for the treatment of first-line mNSCLC, according to the results of the POSEIDON phase III RCT involving 675 individuals with mNSCLC. Individuals were simulated to undergo either T + D + CT for up to four 21-day cycles, followed by durvalumab once every 4 weeks until disease progression or unacceptable toxic effects and one additional tremelimumab dose, or CT for up to six 21-day cycles (with or without pemetrexed maintenance; all groups) in the analysis.

**Main outcomes and measures:** Lifetime costs, quality-adjusted life-years (QALYs), and incremental cost-effectiveness ratio (ICER) were evaluated with a willingness-to-pay (WTP) threshold of $ 100,000 to $ 150,000 per QALY. The uncertainty of the model was investigated using univariate and probabilistic sensitivity analysis.

**Results:** T + D + CT produced additional 0.36 QALYs with additional costs of $ 217,694, compared to CT, giving rise to ICERs of $ 608,667.86/QALY. The univariate sensitivity analysis demonstrated that the outcomes were most sensitive to the cost of durvalumab. Other variables with a large or moderate influence were the utility of progression-free survival state, utility of progressive disease state, and cost of tremelimumab. Probability sensitivity analysis revealed that T + D + CT had a 0% probability of cost-effectiveness in individuals with mNSCLC at a willingness-to-pay threshold of $ 100,000 to $ 150,000 per QALY.

**Conclusion and relevance:** In this model, T + D + CT was estimated to be less cost-effective than CT for patients with mNSCLC at a WTP threshold of $ 100,000 to $ 150,000 per QALY in the United States. When new combination therapies with remarkable effect become pivotal in the first-line treatment, the price reduction of durvalumab and tremelimumab may be necessary to achieve cost-effectiveness in future possible context.

## Highlights

Question: Is T + D + CT a cost-effective first-line treatment for mNSCLC from the U.S. payer perspective?
Findings: In our cost-effectiveness analysis of data from the POSEIDON phase III randomized clinical trial, the QALYs gained in the base case by patients receiving the treatment of T + D + CT were 0.36 years compared with receiving CT, at a cost of $ 608,667.86 per QALY.
Meaning: To the best of our knowledge, this study is the first to synthesize the latest evidence to estimate the economic benefits of tremelimumab. In this model, T + D + CT was assessed as unlikely to be cost-effective compared with CT in mNSCLC at a WTP threshold of $100,000 to $150,000 per QALY in the United States.

## Introduction

Lung cancer remains the deadliest malignant carcinoma in the United States, with approximately 235,760 cases diagnosed and 131,880 deaths each year (Siegel et al., 2022). Up to 85% of carcinoma of the lungs are diagnosed as non-small cell lung cancer (NSCLC) (de Groot et al., 2018) with poor prognosis, and most of these individuals will be diagnosed with advanced or metastatic disease (Reade and Ganti, 2009). Among American patients identified from 2008 to 2014, the 5-year survival rate was 22.7% for newly diagnosed NSCLC and 5.5% for newly diagnosed NSCLC with distant metastasis (NCI, 2018). For patients with metastatic NSCLC (mNSCLC), tremendous progress has been made over the past decade, greatly improving overall survival (OS) and quality of life (Howlader et al., 2020). In particular, the development of immune checkpoint inhibitors, used as monotherapy or in combination with existing chemotherapy (Mok et al., 2019; West et al., 2019; Herbst et al., 2020; Sezer et al., 2021), in the treatment strategies for previously untreated mNSCLC patients has resulted in an unprecedented extension of survival in a subset of these patients (Borghaei et al., 2021; Herbst et al., 2021; Mazieres et al., 2021; Ren, 2021), and a new era in the management of mNSCLC is ongoing.

Durvalumab is a humanized IgG1 monoclonal antibody that selectively blocks the binding of programmed cell death ligand 1 (PD-L1) to programmed cell death 1 (PD-1) and CD80 and restores anticancer immunity (Stewart et al., 2015; Akinleye and Rasool, 2019). Tremelimumab is a humanized IgG2 monoclonal antibody that selectively blocks cytotoxic T-lymphocyte-associated antigen 4 (CTLA-4) and enhances CD80 and CD86 binding to CD28 (Tarhini and Kirkwood, 2008). Durvalumab, with an early limited course of tremelimumab in combination with chemotherapy, is expected to overcome primary resistance to immune checkpoint inhibitors and could be crucial for early disease control (Gandhi et al., 2018; Asadzadeh et al., 2020; Paz-Ares et al., 2021). The POSEIDON randomized clinical trial (Johnson et al., 2022) have shown a substantial improvement in progression-free survival (PFS) (6.2 months *versus* 4.8 months, hazard ratio [HR], 0.72; 95% CI, 0.60 to 0.86; *p* = 0.0003) and OS (14.0 months *versus* 11.7 months, 0.77; 95% CI, 0.65 to 0.92; *p* = 0.0030) for the first-line treatment of individuals with mNSCLC receiving tremelimumab with durvalumab in combination with chemotherapy (T + D + CT) when compared with chemotherapy (CT). The incidence of treatment-associated adverse events (AEs) with grade ≥3 was similar between the two arms (51.8% vs. 44.4%) (Johnson et al., 2022), indicating that T + D + CT has good safety and efficacy in previously untreated mNSCLC. Tremelimumab plus durvalumab plus platinum-based chemotherapy was approved for NSCLC by the U.S. National Comprehensive Cancer Network (NCCN) in December 2022 and has become a new first-line standard of care choice. Considering the cost-effectiveness of medical decisions can help decision makers and clinicians optimize the allocation of limited medical resources. However, its economic efficiency has not yet been determined.

This study aimed to estimate the cost-effectiveness of T + D + CT *versus* CT in the first-line setting for mNSCLC individuals from the perspective of U.S. payers.

## Materials and methods

### Participants and interventions

We extracted basic clinical data from the phase III, open-label, randomized, global POSEIDON trial (Johnson et al., 2022). Patients with EGFR/ALK wild-type mNSCLC who had not received systemic treatment before were eligible. Based on the POSEIDON study, two types of first-line treatment options were simulated in the model: tremelimumab 75 mg for cycles first to fourth, and cycle sixth, plus durvalumab 1,500 mg for cycles first to sixth and platinum-based chemotherapy for cycles first-fourth, followed by durvalumab maintenance therapy from cycle seventh until disease progression (T + D + CT), or platinum-based chemotherapy (CT) for four cycles. Chemotherapy options included cisplatin (75 mg/m^2^ on day 1) plus gemcitabine (1000 mg/m^2^ on days 1 and 8) for patients with squamous histology and cisplatin (75 mg/m^2^ on day 1) plus pemetrexed (500 mg/m^2^ on day 1) for four cycles, with pemetrexed maintenance therapy until disease progression for patients with non-squamous histology. Crossover was not permitted in the POSEIDON trial. As observed in the POSEIDON study (Johnson et al., 2022), 40.8% of individuals in the T + D + CT group and 60.2% of individuals in the CT group received follow-up treatment (Supplementary Table S1). Since the POSEIDON study did not provide any specific details about the follow-up therapy, we assumed that both groups were given either pembrolizumab or docetaxel as a second-line treatment, as immunotherapy and cytotoxic chemotherapy are currently the most frequently utilized treatments.

### Model construction

A Markov model was implemented using TreeAge Pro 2022 software (TreeAge, Williamstown, Massachusetts, United States) to evaluate the costs and effectiveness of mNSCLC, and statistical analysis was performed using R software (version 4.2.1, http://www. r-project.org). Three mutually exclusive health statuses constituted the model structure: PFS, progressive disease (PD), and death (Supplementary Figure S1). Since almost all individuals in both groups died within 200 months in the model simulation, we set the time limit for our analysis to 200 months. One cycle length in this model was defined as 1 month (West et al., 2019). Individuals were partitioned according to the cumulative probabilities of PFS and OS and those stemming from the patient data from the POSEIDON study. We hypothesized that both arms of the patients could receive follow-up therapy until death, when first-line therapy continues until disease progression or unacceptable toxicity occurs. Only direct medical expenditure was considered. Suppose that the corresponding expense is incurred at the beginning of each cycle, there is no cost adjustment for the half cycle. Quality-adjusted life-years (QALYs), total costs, and incremental cost-effectiveness ratios (ICERs) were the main outputs, and we adopted a discount rate of 3% per year for our analysis.

GetData Graph Digitizer software package (version 2.22; http://www. getdata-graph-digitizer.com/index.php) was used to extrapolate the transition probability according to the PFS and OS curves of the POSEIDON trial (Johnson et al., 2022). Pseudo-individual patient data were produced by applying the algorithm deduced by Hoyle et al. (Hoyle and Henley, 2011) to enhance the accuracy of average survival evaluations (Wan et al., 2015). Log-logistic distributions were fitted to the pseudo-individual patient data because they offered optimum fit in light of the Akaike and Bayesian information criterion among survival functions such as exponential, log-normal, gamma, gengamma, Gompertz, Weibull, log-logistic, and distributions were fitted to the data from curves (Supplementary Table S2). The risk of death was determined using an OS curve. The KM and parameter survival distributions for OS and PFS in the model are shown in Figure 2. U.S. life tables were used to assess the background death rates for each age group (Supplementary Table S4) (Arias and Xu, 2022). The virtual patient-level data that we calculated were closely equal to those of the POSEIDON study (Supplementary Figure S2; Supplementary Table S2) (Hoyle and Henley, 2011).

### Costs estimates

Costs were evaluated from the perspective of U.S. payers. Health resource use and direct medical expenditures were considered, including those associated with drug acquisition, drug administration, disease management, and treatment-related AEs (Supplementary Table S3). The drug dosage was calculated according to a body surface area of 1.86 m^2^ (Goulart and Ramsey, 2011). We only considered severe AEs (grade ≥3) with an incidence of greater than 5% in the model, including anemia, neutropenia and thrombocytopenia (Johnson et al., 2022).

Drug prices in this analysis were extracted from the Centers for Medicare and Medicaid Services (https://www.cms.gov/ Accessed 29 December 2022) (CMM, 2022) and Drugs.com Drug Information Database (https://www.drugs.com/price-guide/ Accessed 29 December 2022) (Drugs.com, 2022). The costs of AEs treatment, administration, best supportive care, terminal palliative care, and disease management (involving hospitalization costs, computed tomography expenses, and laboratory examination fees) were also extracted from previously published databases (Georgieva et al., 2018; Lin et al., 2020). We assumed that after the occurrence of AEs, patients were treated only in the first cycle, and the cost of AE occurred only once. The U.S. consumer price index was applied to adjust the costs for inflation to reflect USD 2022. We used the Medical-Care Inflation dataset in Tom’s Inflation Calculator to inflate the costs to 2022 values (Medical-care-inflation, 2022).

### Health-state utilities

QALYs are defined as integrating life span with health-related quality of life, usually called utilities (health state values from 0, indicating death, to 1, indicating full health) (Whitehead and Ali, 2010). The health utility scores for PFS, PD, and death status were extracted from previously published studies and were 0.673, 0.473, and 0, respectively (Nafees et al., 2008). AEs that resulted in disutility values and cost changes were also calculated in our analysis (Nafees et al., 2008; Tolley et al., 2013; Westwood et al., 2014; Lin et al., 2020). The decline in the overall QALY related to all AEs was applied in the first cycle of the model (Su et al., 2021). All the parameters associated with the utilities are listed in Supplementary Table S3.

### Univariate and probabilistic sensitivity analyses

Sensitivity analyses were performed to explore the influence of the parameter uncertainty on the outcomes. Clinical parameters in the univariate sensitivity analysis varied within reasonable limits, derived from the confidence intervals or assumptions with a 20% variance from the baseline value, as shown in the tornado diagram. We conducted 1,000 Monte Carlo simulations to proceed with the probability sensitivity analysis through simultaneous and random preset parameter variations in accordance with specific distribution patterns (see Supplementary Table S3).

In light of real-world performance, there is no possibility that the price of durvalumab, tremelimumab, and pembrolizumab will increase; therefore, only the influence of the price slide on ICERs was examined. Scatter plots and cost-effectiveness acceptability curves (CEACs) were applied to analyze the cost-effectiveness of different options with different willingness-to-pay (WTP) thresholds (Rabin and de Charro, 2001; Lin et al., 2020). As there is no explicit WTP threshold in the U.S., reference was made to the recommended WTP threshold range of the Institute for Clinical and Economic Review to interpret the results ($ 100,000–150,000/QALY) (West et al., 2019). We chose a gamma distribution for the cost parameters, a beta distribution for proportion, probability, and preference value parameters.

## Results

The median PFS and interim OS values obtained in our simulation were consistent with those obtained in the POSEIDON study (Supplementary Table S2). Our model assessed a median PFS of 6.2 months in the T + D + CT arm and 4.8 months in the CT arm. This is similar to data derived from the POSEIDON study. Our models assessed the interim OS analysis of 13.9 and 11.6 months for the T + D + CT and CT groups, respectively. It compared with OS of 14.0 and 11.7 months in the T + D + CT and CT arm, respectively based on the POSEIDON trial.

### Base case results

Within a 200-month horizon based on the Markov model, the total costs were $ 352,291 and $ 134,598 for the T + D + CT and CT arms, respectively. The T + D + CT arm yielded 1.22 QALYs and the CT arm yielded 0.87 QALYs. Therefore, individuals in the T + D + CT arm cost an additional $ 217,694 and produced an increase of 0.36 QALYs, giving rise to an ICER of $ 608,667.86 per QALY, above the pre-set WTP threshold ($ 100,000–150,000/QALY), indicating that the treatment of T + D + CT was not economical compared to CT (Table 1).

**TABLE 1:** Base-case results of the model.

Arm	Costs, US $	△Costs, US $	QALYs	△QALYs	ICER US $/QALY
CT	134,598	—	0.87	—	—
T + D + CT	352,291	217,694	1.22	0.36	608,667.86

ICER, incremental cost-effectiveness ratio; QALY, quality-adjusted life-years.

### Sensitivity analysis

**FIGURE 1:**
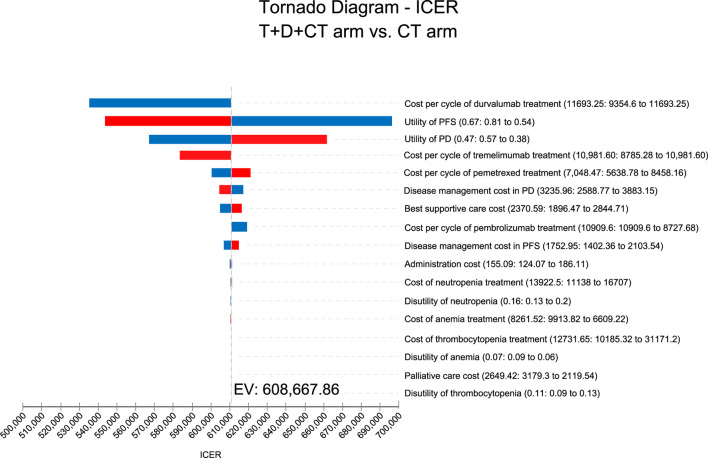
Tornado diagram for univariate sensitivity analyses.

As shown in the tornado diagram for patients with mNSCLC in Figure 1, the parameter that most affected the ICER was the cost of durvalumab. Other variables with large or moderate influences were the utility of the PFS state, utility of the PD state, and cost of tremelimumab. There is no intersection between the generated ICER and WTP when all the parameters vary within the corresponding ranges, confirming that the model results are generally robust.

**FIGURE 2:**
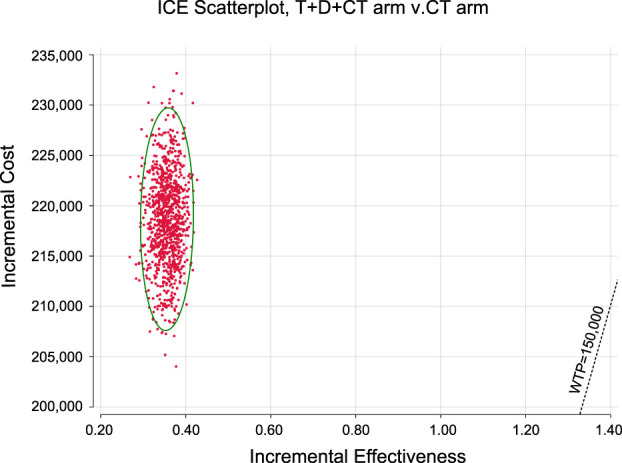
Incremental cost-effectiveness scatter plot diagram for serplulimab arm *versus* placebo arm.

**FIGURE 3:**
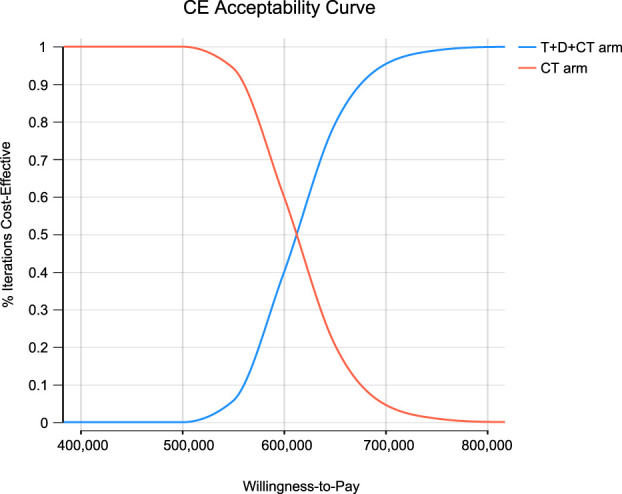
The cost-effectiveness acceptability curves for probabilistic sensitivity analyses.

Monte Carlo simulation of 1,000 individuals revealed that the scatter points were located in the first quadrant of the coordinate axis, indicating that T + D + CT may produce more QALYs but at a higher cost. When WTP was set at $ 100,000–150,000, all scatter points were located above the WTP line (Figure 2). As shown in Figure 3, CEACs indicated that T + D + CT had a 0% probability of cost-effectiveness when the designated WTP threshold was $100,000–150,000 per QALY *versus* CT.

## Discussion

To our knowledge, this study is the first to estimate the economic benefits of tremelimumab using a mathematical modeling method. Based on our model outcomes, our baseline analysis indicated that T + D + CT cost more ($ 352,291 vs. $ 134,598) and produced more health results than CT (1.22 vs. 0.87 QALYs), giving rise to ICERs of $ 608,667.86/QALY. Probability sensitivity analysis indicated that combination therapy is unlikely to be an effective and economical scheme for mNSCLC individual, which was well above the pre-specified WTP threshold ($ 100,000–150,000/QALY) *versus* CT.

The costs per cycle of durvalumab and tremelimumab both had substantial impacts on sensitivity analyses. Although the most sensitive variable changed within a certain range (range, $9,354.60-$11,693.25 per cycle of durvalumab; $8,785.28-$10,981.60 per cycle of tremelimumab), the ICERs were still greater than $150,000 per QALY, which was not considered as cost-effective. The new combination therapy with significant efficacy could keep patients under expensive treatment for longer, and its high cost cannot be neglected. Therefore, lowering the prices of durvalumab and tremelimumab is considered the most practical measure to achieve cost-effectiveness in first-line T + D + CT. Meanwhile, the combination of T + D + CT seems to only offer minor rather than significant enhancements in OS (14.0 months compared to 11.7). In the United Kingdom, upon the introduction of the purchaser/provider model, influential parties involved set a minimum requirement of an additional 3 months of OS for a new therapy to be deemed as a significant advancement and deserving of additional funding (Ferguson et al., 2000). Therefore, there are legitimate concerns surrounding the overall value of new cancer medications that offer limited health benefits but come with continuously escalating prices (Cohen, 2017). Furthermore, payers commonly express concerns about the reliability of PFS as a predictor of OS in solid tumors. As a result, payers tend to favor OS data over PFS data (Cortazar et al., 2014; Prasad et al., 2015; Paoletti et al., 2020), Therefore, European payers and their advisors aimed to reduce the importance of PFS when evaluating the worth of novel cancer medications (Wild et al., 2016). The parameters that also had a large influence on our model were the utilities of the PFS and PD states. The utility value adopted in the analysis refers to published NSCLC patient health utility value data (Nafees et al., 2008). To clarify the impact of health utility value on our model, the ranges of variables were defined for each utility value in the sensitivity analysis (range, 0.5384–0.8076 for the PFS utility; range, 0.3784–0.5676 for the PD utility). The findings illustrated that the upper and lower limits of utility value did not make the combination treatment of T + D + CT cost-effective. The sensitivity analysis focuses on the uncertainty of the model parameters, which confirms the robustness of our model.

The introduction of PD-L1 inhibitor plus chemotherapy combined with quantification of the addition of CTLA-4 inhibitor in mNSCLC in the first-line setting has important clinical implications because of the potentially large population that could benefit from this innovative combination therapy. Although the approval of T + C + CT represents another major step toward supplying a more successful first-line therapy strategy for mNSCLC, it is worth noting that from an individual perspective, the high pricing of antitumor agents may expose individual carcinoma patients to serious risk of economic toxicity, caused by the financial burden of self-paying medical costs that are not included in health insurance (Carrera et al., 2018). Economic toxicity has been shown to result in discontinuation, delay, and abandonment of treatment for patients (Carrera et al., 2018). It is just as important for healthcare systems to ensure that individuals have access to novel treatments to minimize financial harm (de Souza and Conti, 2017). However, negotiating lower prices of durvalumab and tremelimumab through trade-offs of drug pricing and coverage could be an effective and appropriate way to increase cost-effectiveness. The approval of new drugs based solely on survival benefits is challenging. This is the reason for considering different economic factors in different regions in the process of drug approval to promote rational clinical drug use and provide a reference for evidence-based decision-making by medical insurance departments.

Several cost-effectiveness studies on PD-(L)1 plus chemotherapy plus CTLA-4 blockade as a first-line therapy for NSCLC have been conducted (Peng et al., 2021; Polyzoi et al., 2022), with the aim of combining the persistence of combined immunotherapy with the initial beneficial effect of chemotherapy. Based on the data published in the CheckMate 9LA trial (Paz-Ares et al., 2021), Polyzoi et al. indicated that compared with platinum-doublet chemotherapy alone in first-line advanced NSCLC, nivolumab plus ipilimumab plus platinum doublet chemotherapy yielded incremental 0.80 QALYs and ICERs of $202,275/QALY, again unlikely to be cost-effective from the U.S. perspective at a WTP of $150,000/QALY (Polyzoi et al., 2022), which was consistent with our analysis. Thus, when novel combination treatments with significant efficacy are key to first-line therapy, lowering the cost of these agents might be crucial to achieve cost-effectiveness. Tailoring treatments for individual patients or lowering the costs of immune checkpoint inhibitors can enhance economic benefits. These results may inform clinicians when making the best decisions regarding mNSCLC treatment.

Patients’ age, health status, or disease severity can significantly affect functional independence in mNSCLC (Camps et al., 2009). Patient-reported health-related quality of life (HRQoL) offers a comprehensive assessment of health, wellbeing and daily functioning (Cella and Patel, 2008), HRQoL can be expressed as a health state utility value ranging from 0 (death) to 1 (full health) (Paracha et al., 2018). Therefore, the conditions of patients are mainly reflected by the differences in health state utility values. To avoid selection bias due to the study focusing only on a small number of patients who meet specific inclusion and exclusion criteria, and utility values disproportionately affect outcome stability, multiple researches were referred to broaden the range of utility values in the sensitivity analyses. Health state utilities were varied within ±20%. The results indicate that the utility value does not have a significant impact on the findings and both upper and lower limits of utility value indicate that T + D + CT strategy is not cost-effective in the first line therapy for mNSCLC patients. Thus, this limitation is alleviated to some extent, namely, that the data of our study may not accurately represent the broader population who would be prescribed the new treatment. However, the shortcomings of randomized controlled trial used in our study, particularly “standardised trials” designed for new drug approval applications, are widely recognised, and future models need to take rational design further into account.

Conducting sensitivity analyses aims to thoroughly investigate the impact of parameter uncertainty and model structure on outcomes, considering the disparity between clinical trials and the real-world. In cost-effectiveness analyses, the reliability of the study data is uncertain because it has to be acknowledged that clinical trial data may not be able to capture the real-world situations that patients face when taking a new treatment; nonetheless, this limitation may not have significant influences, as demonstrated by the results in the sensitivity analyses, implying that enhancing the certainty of these estimates may not yield significant value. Clinical parameters in the univariate sensitivity analyses varied within ±20%. According to specific distribution patterns, a set of 1,000 Monte Carlo simulations were carried out to perform probability sensitivity analyses with random and simultaneous preset parameter variations. Both univariate and probability sensitivity analyses revealed that our results remained robust, and more studies into the real-world efficacy of therapy in previously untreated mNSCLC patients is warranted to further clarify comparative cost-effectiveness. Additionally, utilizing real-world research data could assist with validating the model over time.

This study had some limitations. First, the survival curves must be extrapolated to acquire complete survival results because of the short follow-up period of the POSEIDON study (Johnson et al., 2022). The results of the actual survival curves could not be entirely fitted to the reconstructed survival curves. Nevertheless, the objective of adjusting the transition probability was to approach the actual results as closely as possible. Second, owing to the short quality of life data in the model to calculate the health state utility values that were extracted by referring to published literature, the disutility values of AEs were considered for correction, including only ≥3 grade AEs, which may cause an underestimation or overestimation of utility values. Our sensitivity analysis revealed that the utility value in the PFS state had a significant effect on ICERs. However, when the utility value varied by ±20%, ICERs remain exceeded the WTP threshold, confirming the robustness of the results. Third, the time horizon was set at 200 months to make sure that the model still runs after over 99% of cohort participants came to the death outcome, and it was a period expected to cover the entire lifespan of patients. At this point, it is believed that very few patients survive after 200 months, and survivors have little impact on the outcome. Fourth, the research simulated findings originating from a randomized clinical trial but not from a prospective real-world study, which is inevitably affected by uncertainty. Implemented a range of sensitivity analyses to assess uncertainty; however, the long-term benefits of the addition of a limited course of CTLA-4 inhibitors and PD-L1 inhibitors to chemotherapy remain an open question. The more mature the available data, the more stable the model. This model can verify long-term survival data and serve as a basis for treatment recommendations in patients with mNSCLC.

Payer’s perspective was used instead of the societal perspective (e.g., the time and transportation costs associated with receiving chemotherapy), and this decision was made because the study was primarily focused on evaluating the financial burden on the healthcare system or insurance provider rather than considering broader societal impacts. Second, the payer’s perspective is more applicable in this particular case due to other factors such as the availability of data or the research question being addressed. Additional, the analysis was preformed from the payer perspective, but indirect costs (e.g., productivity losses) were not included. Although it is important to take into account the societal expenses when making decisions associated with allocating resources, the expected influence of these costs would be significantly lower compared to direct medical expenses like hospitalizations and product costs. Still, it is necessary to acknowledge the limitations and potential biases associated with our decision.

In looking to the future, as the field of personalized medicine advances, it has become clear that patients with previously untreated mNSCLC or other cancers require novel therapeutic technologies. However, given the significant increase in drug pricing in recent years, it is crucial to ensure that these novel treatments are utilized appropriately by identifying and targeting those individuals who are most likely to benefit. In today’s landscape, expensive medications encounter few obstacles in terms of receiving coverage and being adopted by doctors. Nevertheless, the procedure of approving novel drugs and integrating them into therapeutic formulary and guideline could ultimately require decision-makers and physicians to more definitely confront trade-off between medical cost and clinical benefits. Moreover, the willingness of payers, patients, and other stakeholders to cover the expenses of costly cancer treatments will set a fresh benchmark for cost-effective healthcare. Data derived from this research and others like it offer a initiating point for such discussions. In order to increase the widespread and appropriate adoption of cost-effectiveness analysis, it needs to be integrated into a comprehensive framework that takes into account the extra dimensions contributing to societal value. Cost-effectiveness analysis could integrate these dimensions, provided that a consensus on these dimensions can be reached and they can be accurately measured. While having reservations about its near-term feasibility, integrating cost-effectiveness analysis into a broader process could aid in the resolution and legitimization of intricate ethical dilemmas surrounding the distribution of healthcare resources. Going forward, there may be a transition from solely relying on cost-effectiveness analysis to incorporating cost-benefit analyses for healthcare. This would allow for a broader consideration of trade-offs across all social resources, rather than just within the healthcare field. No matter what method is taken, to ensure effective healthcare decisions, various factors must be considered and weighed carefully, such as efficacy, safety, patient satisfaction and preference, resource utilization rate, and social equity. These factors influence how individuals place value on personal health, the health of others, and the healthcare system that serves everyone. In this study, only two treatments, chemotherapy and immunotherapy, were selected. However, it is important to note that there are many other treatment options available for advanced NSCLC, such as radiation therapy, targeted therapy, and other therapeutic approaches in addition to drug chemotherapy. Therefore, pharmacoeconomic evaluation will be further carried out for different treatment schemes for advanced NSCLC in the future, in order to provide more decision-making basis for patients. It is crucial to acknowledge the heterogeneous economic development across countries and take into consideration the WTP when assessing the cost-effectiveness. Given that both durvalumab and tremelimumab exhibit significant impacts on sensitivity analyses in terms of costs per cycle, it is plausible to assume that without reducing drug prices, these interventions may not be more cost-effective than chemotherapy in other countries worldwide. This perspective warrants further investigation and validation in future research.

## Conclusion

From the perspective of the U.S. healthcare system, T + D + CT was estimated to be unlikely to be more cost-effective than CT in previously untreated individuals with mNSCLC at a WTP threshold of $10,000 to $15,000 per QALY. Price reductions remain the most practical solution to achieve a balance between the incremental cost and quality-adjusted survival gain for T + D + CT regimen. In addition, It is recommended that both the clinical outcomes and health-economic implications should be considered in the decision-making process. For patients with good economic conditions, T + D + CT combination therapy can be given priority. For patients with limited economic conditions, chemotherapy can be considered first based on their economic conditions and insurance situation. In the future, intermittent immune combined chemotherapy will be a research direction, and if the efficacy of intermittent immune combined chemotherapy is not significantly different from that of T + D + CT combination therapy, intermittent combination therapy can greatly reduce drug costs and become a cost-effective treatment strategy. On the other hand, ICER is a value that changes over time, and as drug prices decrease, the economic advantages of combination therapy will gradually become apparent. Therefore, this research has certain reference value for policymakers and physicians. However, because of the several limitations in this study, further long-term follow-up data and real-world data are needed.

## Data Availability

The original contributions presented in the study are included in the article/Supplementary Material, further inquiries can be directed to the corresponding author.
