# Supplementary material for: First-line tremelimumab plus durvalumab and chemotherapy versus chemotherapy alone for metastatic non-small cell lung cancer: a cost-effectiveness analysis in the United States
Source: Front Pharmacol. 2023 Jul 20;14:1163381. doi: 10.3389/fphar.2023.1163381 (PMC10398575; doi:10.3389/fphar.2023.1163381)
Supplement: Supplementary file 1 [file Table1.doc]

**Table S1** AIC and BIC for different parameter distributions of PFS and OS

| Distribution | PFS | | | | OS | | | |
| --- | --- | --- | --- | --- | --- | --- | --- | --- |
|  | T + D + CT arm | | CT arm | | T + D + CT arm | | CT arm | |
|  | AIC | BIC | AIC | BIC | AIC | BIC | AIC | BIC |
| exponential | 1526.159 | 1529.979 | 1580.677 | 1584.500 | 2110.703 | 2114.523 | 2098.277 | 2102.100 |
| gamma | 1495.391 | 1503.032 | 1575.759 | 1583.405 | 2105.216 | 2112.856 | 2099.991 | 2107.637 |
| gengamma | 1494.094 | 1505.554 | 1556.584 | 1568.054 | 2094.971 | 2106.431 | 2087.264 | 2098.734 |
| gompertz | 1522.584 | 1530.224 | 1580.42 | 1588.066 | 2112.662 | 2120.302 | 2089.07 | 2096.716 |
| weibull | 1501.601 | 1509.241 | 1579.651 | 1587.297 | 2108.449 | 2116.089 | 2098.903 | 2106.549 |
| Log-logistic | **1490.448** | **1498.089** | **1552.143** | **1559.789** | **2094.129** | **2101.769** | **2082.683** | **2090.329** |
| Log-normal | 1503.433 | 1511.073 | 1554.587 | 1562.233 | 2094.447 | 2102.087 | 2086.439 | 2094.085 |

**Table S2** The median progression-free survival and interim overall survival analysis observed in the POSEIDON trial and estimated by the current cost-effectiveness model

| Treatment | Model | Trial of POSEIDON | Difference |
| --- | --- | --- | --- |
| Median progression-free survival, mo |  |  |  |
| T + D + CT arm | 6.2 | 6.2 | 0 |
| CT arm | 4.8 | 4.8 | 0 |
| Median overall survival, mo |  |  |  |
| T + D + CT arm | 13.9 | 14.0 | 0.1 |
| CT arm | 11.6 | 11.7 | 0.1 |

**Table S3** Model parameters

| Variable | Baseline value (Reference) | Range | | Distribution |
| --- | --- | --- | --- | --- |
|  |  | Minimum | Maximum |  |
| Log-logistic PFS survival model with T + D + CT arm | Shape = 1.551; scale = 6.155 | - | - | - |
| Log-logistic PFS survival model with CT arm | Shape = 1.886; scale = 4.802 | - | - | - |
| Log-logistic OS survival model with T + D + CT arm | Shape = 1.277; scale = 14.118 | - | - | - |
| Log-logistic OS survival model with CT arm | Shape = 1.577; scale = 11.748 | - | - | - |
| Subsequent chemotherapy proportions of T + D + CT arm | 0.317 ^[1]^ | 0.2536 | 0.3804 | Beta |
| Subsequent immunotherapy proportions of T + D + CT arm | 0.091 ^[1]^ | 0.0728 | 0.1092 | Beta |
| BSC in T + D + CT arm | 0.592 ^[1]^ | 0.4736 | 0.7104 | Beta |
| Subsequent chemotherapy proportions of CT arm | 0.362 ^[1]^ | 0.2896 | 0.4344 | Beta |
| Subsequent immunotherapy proportions of CT arm | 0.240 ^[1]^ | 0.1920 | 0.2880 | Beta |
| BSC in CT arm | 0.398 ^[1]^ | 0.3184 | 0.4776 | Beta |
| Grade ≥3 AEs incidence in T + D + CT arm | | | | |
| Anemia | 0.173 ^[1]^ | 0.1384 | 0.2076 | Beta |
| Neutropenia | 0.161 ^[1]^ | 0.1288 | 0.1932 | Beta |
| Thrombocytopenia | 0.055 ^[1]^ | 0.0440 | 0.0660 | Beta |
| Grade ≥3 AEs incidence in CT arm | | | | |
| Anemia | 0.204 ^[1]^ | 0.1632 | 0.2448 | Beta |
| Neutropenia | 0.120 ^[1]^ | 0.0960 | 0.1440 | Beta |
| Thrombocytopenia | 0.051 ^[1]^ | 0.0408 | 0.0612 | Beta |
| Utility | | | | |
| Progression-free disease | 0.673 ^[2]^ | 0.5384 | 0.8076 | Beta |
| Progressed disease | 0.473 ^[2]^ | 0.3784 | 0.5676 | Beta |
| Discount rate (%) | 3 ^[3]^ | 0 | 5 | Fixed |
| AEs disutility | | | | |
| Anemia | -0.073 ^[4]^ | -0.0584 | -0.0876 | Beta |
| Neutropenia | -0.163 ^[2]^ | -0.1304 | -0.1956 | Beta |
| Thrombocytopenia | -0.108 ^[5]^ | -0.0864 | -0.1296 | Beta |
| Patients’ body surface area (m^2^) | 1.86 ^[6]^ | 1.4880 | 2.2320 | Normal |
| Drug cost, US $ | | | | |
| Durvalumab/cycle | 11693.25 ^[7]^ | 9354.60 | 11693.25 | Gamma |
| Tremelimumab/cycle | 10981.60 ^[8]^ | 8785.28 | 10981.60 | Gamma |
| Pembrolizumab/cycle | 10909.60 ^[7]^ | 8727.68 | 10909.60 | Gamma |
| Cisplatin/cycle | 26.73 ^[7]^ | 21.38 | 32.07 | Gamma |
| Gemcitabine/cycle | 68.37 ^[7]^ | 54.70 | 82.05 | Gamma |
| Pemetrexed/cycle | 7048.47 ^[7]^ | 5638.78 | 8458.16 | Gamma |
| Docetaxel/cycle | 65.29 ^[7]^ | 52.23 | 78.34 | Gamma |
| AEs cost, US $ | | | | |
| Anemia | 8261.52 ^[3]^ | 6609.216 | 9913.824 | Gamma |
| Neutropenia | 13922.50 ^[3]^ | 11138 | 16707 | Gamma |
| Thrombocytopenia | 12731.65 ^[3]^ | 10185.32 | 15277.98 | Gamma |
| Best supportive care cost, US $ | 2370.59 ^[3]^ | 1896.472 | 2844.708 | Gamma |
| Palliative care cost, US $ | 2649.42 ^[3]^ | 2119.536 | 3179.304 | Gamma |
| Administration cost, US $ | 155.09 ^[3]^ | 124.072 | 186.108 | Gamma |
| Disease management cost in PFS, US $ | 1752.95 ^[3]^ | 1402.36 | 2103.54 | Gamma |
| Disease management cost in PD, US $ | 3235.96 ^[3]^ | 2588.768 | 3883.152 | Gamma |

References

[1] Johnson ML, Cho BC, Luft A, et al. Durvalumab With or Without Tremelimumab in Combination With Chemotherapy as First-Line Therapy for Metastatic Non-Small-Cell Lung Cancer: The Phase III POSEIDON Study. J Clin Oncol. 2022 : JCO2200975.

[2] Nafees B, Stafford M, Gavriel S, Bhalla S, Watkins J. Health state utilities for non small cell lung cancer. Health Qual Life Outcomes. 2008. 6: 84.

[3] Lin S, Luo S, Zhong L, et al. Cost-effectiveness of atezolizumab plus chemotherapy for advanced non-small-cell lung cancer. Int J Clin Pharm. 2020. 42(4): 1175-1183.

[4] Westwood M, Joore M, Whiting P, et al. Epidermal growth factor receptor tyrosine kinase (EGFR-TK) mutation testing in adults with locally advanced or metastatic non-small cell lung cancer: a systematic review and cost-effectiveness analysis. Health Technol Assess. 2014. 18(32): 1-166.

[5] Tolley K, Goad C, Yi Y, Maroudas P, Haiderali A, Thompson G. Utility elicitation study in the UK general public for late-stage chronic lymphocytic leukaemia. Eur J Health Econ. 2013. 14(5): 749-59.

[6] Goulart B, Ramsey S. A trial-based assessment of the cost-utility of bevacizumab and chemotherapy versus chemotherapy alone for advanced non-small cell lung cancer. Value Health. 2011. 14(6): 836-45.

[7] The Centers for Medicare & Medicaid Services. Available at: https://www.cms.gov/ (Accessed December 29, 2022) .

[8] The Drugs.com Drug Information Database. Available at: https://www.drugs.com/price-guide/ (Accessed December 29, 2022) .
