# Supplementary material for: First-line tremelimumab plus durvalumab and chemotherapy versus chemotherapy alone for metastatic non-small cell lung cancer: a cost-effectiveness analysis in the United States
Source: Front Pharmacol. 2023 Jul 20;14:1163381. doi: 10.3389/fphar.2023.1163381 (PMC10398575; doi:10.3389/fphar.2023.1163381)
Supplement: Supplementary file 2 [file Image1.pdf]

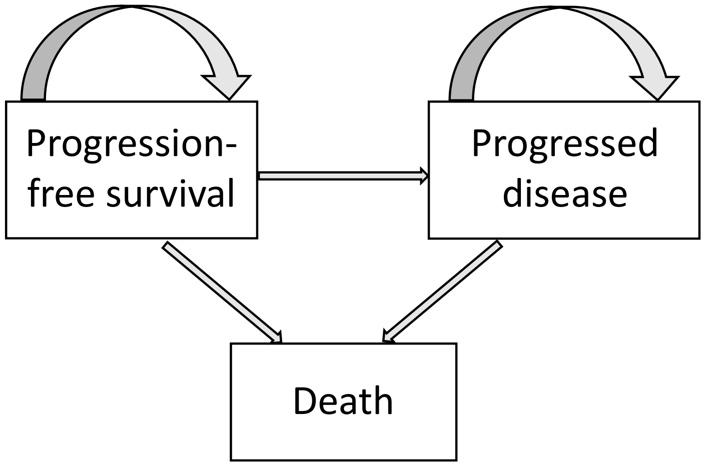

Figure S1 Partitioned survival model structure

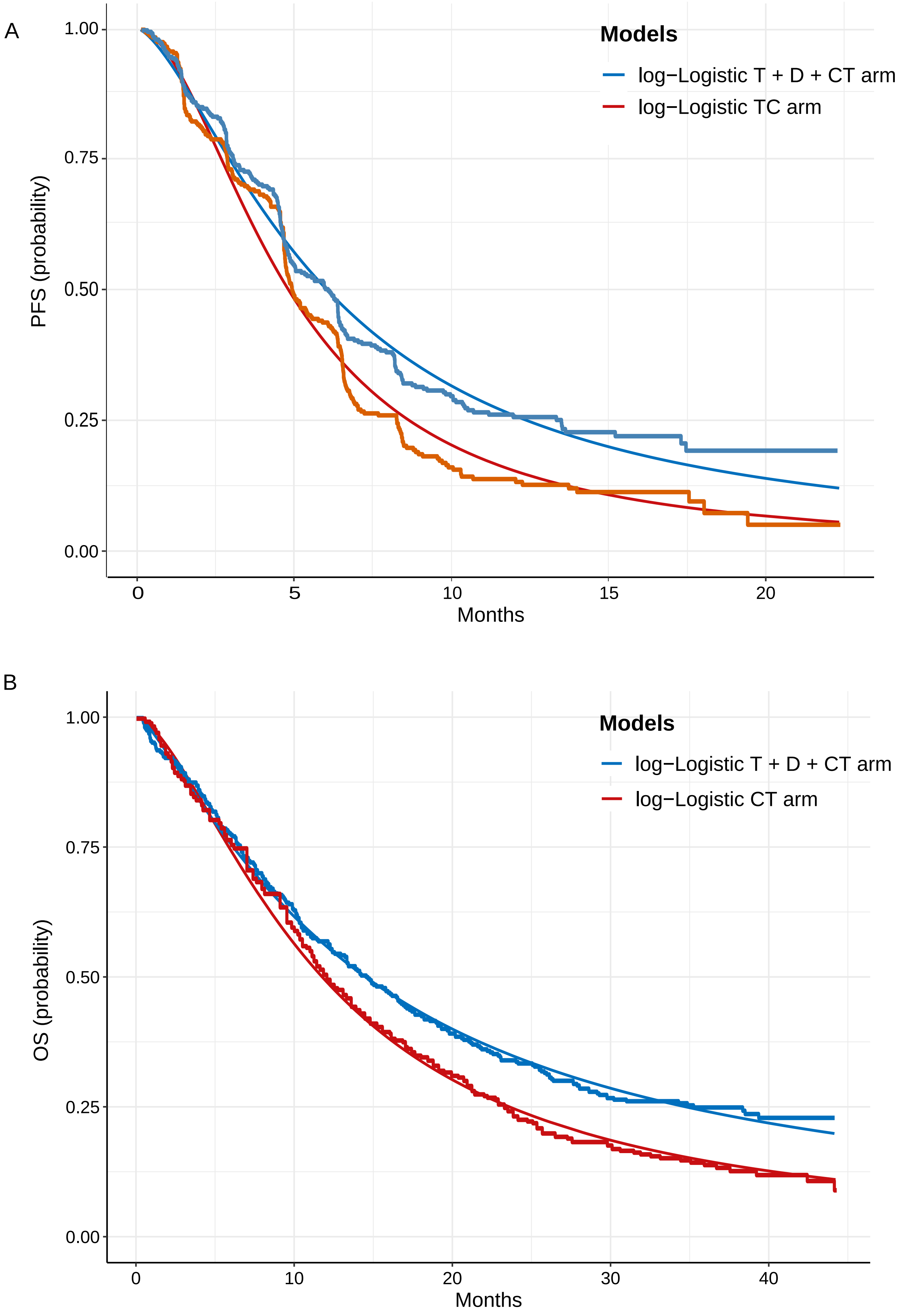

Figure S2 PFS curves for the original trial and model estimated data (A) and OS curves for the original trialand estimated data (B)
